# A crowdsourcing approach for reusing and meta-analyzing gene expression data

**DOI:** 10.1038/nbt.3603

**Published:** 2016-06-20

**Authors:** Naisha Shah, Yongjian Guo, Katherine V Wendelsdorf, Yong Lu, Rachel Sparks, John S Tsang

**Affiliations:** 1 Systems Genomics and Bioinformatics Unit, National Institute of Allergy and Infectious Diseases, National Institutes of Health, Bethesda, Maryland USA; 2 Office of the Chief Laboratory of Systems Biology, National Institute of Allergy and Infectious Diseases, National Institutes of Health, Bethesda, Maryland USA

**Keywords:** Data integration, Data mining, Computational platforms and environments

To the Editor:

Advances in high-throughput technologies have led to a rapid increase in the amount of data generated on a molecular, cellular and organismal scale^1,2^. The reuse and meta-analysis of large-scale data from multiple independent studies can increase the statistical power to obtain new and robust biological insights, compared with the analysis of any one study, and may serve as a productive starting point for informing the design of experiments^3^. Previous studies have successfully combined publicly available data from published studies to both reposition drugs^4^ and identify robust gene-expression signatures of transplant rejection^5^, infection status^6,7^, tumor subtypes and cancer progression^8^. However, these meta-analysis approaches are not trivial, often requiring study-related information that is not always available, as well as computational and statistical expertise that could discourage direct, hands-on participation of many biologists.

Here we present OMics Compendia Commons (OMiCC) (https://omicc.niaid.nih.gov), a freely available tool, aimed at biologists with limited bioinformatics training, that uses a crowdsourcing approach to help overcome some of these challenges. OMiCC enables the broader biomedical research community to generate and test hypotheses through reuse and (meta-) analysis of existing data sets. Annotations, metadata and components of cross-study data compendia created by users are stored and made available to other users of the platform so that they may build on previous analyses and contribute their own annotations and analysis designs. In this way, OMiCC may help bring down barriers across communities and encourage a culture of sharing and openness in biomedical research.

Millions of gene expression profiles reside in public databases^1,2^. These data could potentially be used to generate, assess or replicate hypotheses, even if the experiments were not originally designed to answer the same research questions. For example, data for evaluating the effect of a drug (in which drug-treated versus untreated subjects are compared) could be used to investigate the effects of gender on drug treatment. In addition, meta-analysis approaches^9^ will become increasingly effective for drawing robust conclusions from similar data sets generated from independent studies.

However, the wealth of information available in public databases remains largely untapped, particularly by experimental biologists. One reason for this is that the steps involved in retrieving, processing and analyzing these data can be computationally and statistically complex for many biologists. Numerous resources have been created to enable the reuse and analysis of large-scale expression data (Supplementary Note 1), but they are generally limited to one or a subset of analytical steps, and therefore additional programming is still required for most workflows. Although commercial software has been developed to address some of these limitations, the algorithms are often proprietary, which makes incorporating external data into any analysis difficult, if not impossible. Furthermore, fee-based services could limit the size and diversity of the user community; less well-funded groups and research areas, as well as organizations from developing countries, tend to have less access.

Another major barrier for both experimental and computational biologists alike is that structured, meta-information critical for data reuse and cross-study analyses is typically not readily available. It is often necessary to determine which samples from a study can be grouped, which groups can be meaningfully compared (e.g., a particular type of tumor samples versus normal), and what groups can be collated or compared within and across studies. Constructing such sample groups and comparison pairs requires biological expertise specific to the biological domain of the study; doing so *en masse* for all available studies is thus enormously time-consuming and challenging.

OMiCC provides programming-free capabilities for the (meta-) analysis of public gene-expression data sets. It was also designed to serve as a 'commons' for engaging diverse segments of the biomedical research community to help build a comprehensive and reusable repository of meta-information (e.g., sample groups, pairs and their annotations)—essential building blocks for constructing data compendia and performing meta-analyses (Fig. 1a). OMiCC can further serve as an educational tool to help students learn new biology by exposing them to hands-on exploration of large-scale data sets. This didactic mission is particularly important as biology is increasingly dominated by 'big data'-driven approaches.

**Figure 1:**
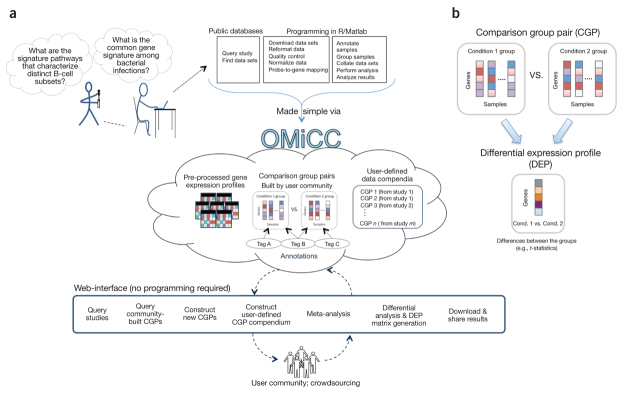
Overview of OMiCC (**a**) OMiCC has two main aims: first, to directly empower biomedical researchers to address biological questions and develop hypotheses through hands-on reuse, integration and (meta-) analyses of publicly available large-scale gene expression data sets across one or more studies without performing programming; and second, to serve as a community, crowdsourcing resource to enable meta-information and data-compendia component reuse. OMiCC comprises two major components: first, a database containing pre-processed gene expression data sets from GEO, meta-information authored by the user community (e.g., CGPs; see (**b**) below for details) and user-defined compendia (a collection of CGPs from one or more studies); and second, a web interface that interacts with the OMiCC database and enables users to create and (meta-) analyze gene expression compendia across studies and platforms. It also enables a user to download collated data sets and analysis results for further analysis outside of OMiCC, using other tools. (**b**) OMiCC enables the creation, annotation and sharing of CGPs, each of which comprises two collections of gene expression profiles from a study. For each CGP, OMiCC computes the differential expression profile (DEP) reflecting the gene-expression difference for all genes/probes between the two groups in the CGP. Sample groups and CGPs are permanently stored on the OMiCC server and can be shared among users for use in different projects and thus can be used as building blocks of compendia.

More than 26,000 pre-normalized and quality-checked human and mouse studies comprising ∼690,000 expression profiles from the Gene Expression Omnibus (GEO) (Supplementary Note 2) are now accessible through OMiCC. A core feature is the ability to easily create, annotate and share comparison group pairs (CGPs; Fig. 1b). A CGP comprises two collections (called sample groups) of gene expression profiles from a study, for example, blood transcriptomes of diabetic patients and of healthy controls. OMiCC provides easy-to-use interfaces for constructing sample groups and CGPs and for annotating them using medical subject headings (MeSH)^10^, a standardized biomedical vocabulary used by PubMed, so that the resulting annotations are more easily interpretable and reusable by the community. Once a CGP is formed, OMiCC can compute significantly differentially expressed genes and a differential expression profile (DEP) capturing the differences in expression values for all genes between the sample groups (Fig. 1b). In contrast to approaches that use only statistically significant differentially expressed genes for comparison among CGPs^4,11^, DEPs can be collated across CGPs spanning one or more studies to form a data matrix operable by existing analysis tools and algorithms, including clustering and gene set enrichment analysis^12^. Assessing expression patterns using DEPs instead of individual expression profiles can also potentially help mitigate study-specific effects, thereby improving data comparability across studies^11^.

Sample groups and CGPs are stored permanently on the OMiCC server and can be made available to the broader community of users. This crowdsourcing approach can thus harness the collective biological expertise of the research community to create structured, reusable content. For example, a researcher working on juvenile idiopathic arthritis would be more aware of disease subtypes, such as oligoarticular, polyarticular and systemic juvenile idiopathic arthritis than non-experts, and thus, would be more able to accurately and rapidly annotate and construct sample groups and CGPs. Over time, this approach could amass a large number of biologically relevant and publicly searchable CGPs (and the corresponding sample groups) that may aid future research.

To encourage the creation and sharing of reusable CGPs by users, OMiCC tracks and publicizes, if the user permits, the number of sample groups and CGPs a user has shared with the community. We also encourage users to share information about their scientific expertise on user profiles by providing links to their homepages, professional social network profiles and PubMed search terms for accessing their publications. Such information could be helpful for the community to gauge whether the contents shared by a user match well with their scientific expertise. OMiCC currently displays the usage statistic of sharable contents (e.g., the number of compendia a CGP is being used in). Together, these features can help users share and reuse sample groups and CGPs.

Data processing and computation in OMiCC are fully automated, but if desired, computational and statistical parameters can be customized by means of the web interface (Supplementary Note 2). Once a DEP data matrix is created, the user can also generate basic visualizations and export the underlying raw data for analysis outside of OMiCC using other tools, such as GenePattern^13^, GENE-E^14^ and R/Bioconductor^15^ (GENE-E can be launched directly from OMiCC with data pre-loaded). To help ensure reproducibility, OMiCC tracks computational runs and associated parameters; analysis results can also be shared with the community using publicly accessible web links.

To illustrate how OMiCC can enable biologists to use public data to address real-world questions, we describe in Box 1 a step-by-step example involving the meta-analysis of inflammatory bowel disease (IBD) across multiple independent studies to obtain and validate robust gene expression and pathway signatures.

Although OMiCC was designed to allow non-specialists to carry out a range of computational analyses, accurate and robust interpretation of results will require a certain degree of statistical expertise. To support the didactic mission of the platform, the OMiCC website provides a tutorial containing a step-by-step guide and several videos to help researchers use the platform. The tutorial also contains background information on and discussions of relevant analysis issues and statistical concepts, such as platform selection, hypothesis testing, multiple testing correction and meta-analysis. In addition, OMiCC has a context-sensitive, 'take-a-tour' feature in some key pages (e.g., CGP and data compendia creation) to guide users interactively through the main workflows.

OMiCC is not intended to replace collaborations with statistical and bioinformatics experts. Users should be aware of the potential pitfalls with cherry-picking results and with multiple testing after applying different statistical tests, analysis parameters and significance cutoffs to assess a question using the same underlying data sets. Furthermore, statistical expertise will likely be needed on certain issues, such as how to interpret meta-analysis results from data sets with very different sample sizes and how to handle potential technical and biological heterogeneity across studies. We recommend that, when in doubt, users consult with bioinformatics experts on issues, such as multiple-testing correction, appropriate statistical tests to use for determining differentially expressed/signature genes^16^, analysis parameter customization and statistical result interpretation. A discussion forum might be a helpful feature to add in the future to help connect experienced and novice users in the community.

Although OMiCC syncs and pre-processes data from a large number of platforms, many platforms are used in a small number of studies, and some cover only a relatively small number of genes. OMiCC allows restricting searches for studies to certain platforms; to guide platform selection, OMiCC also visually highlights the most popular platforms based on the number of studies that used a platform. In general, restricting analyses to the most popular platforms is a prudent strategy as those platforms tend to be better developed, and the degree of gene overlap among them tends to be substantial.

Batch effects within studies can be prevalent^17^, particularly when using CGPs outside the realm of the original study design; therefore, additional checks should be made by consulting metadata, the original publication and its authors on experimental design and batching information. Ultimately, results should be validated using independent data and experimental follow-up. However, meta-analysis can sometimes mitigate the effect of technical factors, such as experimental batch, on analysis results because these effects tend not to be consistent across studies, and therefore the results are robust if coherent signals can be detected across studies and platforms^5,9^. This was illustrated by our IBD use case (Box 1), where coherent signatures found using a set of 'discovery' studies were convincingly replicated using a collection of independent 'validation' studies.

In summary, OMiCC provides a practical, easy-to-use toolkit and a crowdsourced, community-oriented framework to help democratize public gene-expression data reuse and meta-analysis. We are actively considering ways to further open up the platform so that others in the community can contribute to its future evolution and development (e.g., by providing an application programming interface or making it open source). We envision that as more users take advantage of OMiCC to drive biological hypothesis generation and discovery, more users will create and share sample groups and CGPs with the community. Thus, OMiCC has the potential to grow organically into an increasingly rich resource to help add cross-study, meta-analysis approaches to a biologist's toolbox and thus enable more effective transformation of the increasing amounts of public data into biological insights.

**Author contributions**

J.S.T. conceived and directed the project. K.V.W. coordinated early interface design and performed initial data exploration with help from N.S. and Y.G. N.S., Y.G. and K.V.W. designed user interfaces with inputs from J.S.T.; Y.G. designed database and web software with help from N.S. and K.V.W.; Y.G. implemented database and web software; N.S. and Y.G. designed and implemented data processing pipelines with help from Y.L. and inputs from J.S.T.; N.S., Y.G., R.S. and J.S.T. developed website contents with help from Y.L. N.S. and J.S.T. designed use-case analyses; N.S., R.S. and J.S.T. performed analyses; J.S.T. and N.S. interpreted analysis results; J.S.T. and N.S. drafted the manuscript; J.S.T. finalized the manuscript with inputs from other authors.

Box 1: Meta-analysis of inflammatory bowel disease IBD is a common disease characterized by chronic, relapsing intestinal inflammation and epithelial injury with both genetic and environmental contributions^18^. Using data from 11 studies, a previous meta-analysis of Crohn's disease and ulcerative colitis—two major IBD subtypes—identified genes differentially expressed in disease versus healthy controls^19^. To illustrate how OMiCC facilitates meta-analysis without requiring any computer programming by the user, we performed a meta-analysis of Crohn's and ulcerative colitis (Supplementary Note 3). Using search criteria detailed in Supplementary Note 3, we used OMiCC's search interface to find studies containing tissue samples from Crohn's, ulcerative colitis and healthy subjects. We found four studies, from which four disease-versus-healthy CGPs for Crohn's and ulcerative colitis were constructed using OMiCC's web interface (Supplementary Table 1). Once CGPs were defined, OMiCC automatically mapped probes to genes for all platforms, performed meta-analysis for genes common across the CGPs, and reported results, which included average fold-changes and meta-analysis *P*-values (and corresponding false-discovery rates) reflecting combined signals from the CGPs.

Despite potential technical and biological heterogeneity across studies, OMiCC detected 2,213 and 1,778 genes with higher or lower expression in ulcerative colitis compared with healthy controls, respectively, as well as 1,750 and 1,214 such genes in Crohn's disease (false-discovery rate (FDR) < 0.05; Supplementary Table 2 and Supplementary Note 3). These results are broadly consistent with those reported in a previous meta-analysis of IBD^19^. We next took these differentially expressed genes (those with increased or decreased expression were considered separately) and assessed pathway enrichment using ToppGene^20^ (https://toppgene.cchmc.org/; Supplementary Table 3). As expected, given the infiltration and activation of immune cells, genes with increased expression in both Crohn's and ulcerative colitis are highly enriched for immune and inflammatory processes as well as those associated with cell adhesion (FDR < 0.05). Interestingly, several peroxisome proliferator-activated receptors (PPARγ, PPARα and PPARδ) tended to have lower mRNA levels in Crohn's (all FDR < 0.05; unadjusted *P* = 2.62 × 10^−6^, *P* = 6.67 × 10^−4^ and *P* = 2.7 × 10^−4^, respectively) as well as in ulcerative colitis compared with healthy controls (all FDR < 0.05; *P* = 0, *P* = 1.23 × 10^−3^ and *P* = 3.06 × 10^−3^, respectively). A previous report highlighted PPARγ expression to be lower in ulcerative colitis, but not necessarily in Crohn's patients^21^. Furthermore, other genes from the PPAR signaling pathway (as annotated by the Kyoto Encyclopedia of Genes and Genomes (KEGG)) as well as from various metabolic and catabolic processes, including those of lipids (Gene Ontology (GO) term GO:0006629), organic acids (GO:0006082), and small molecules (GO:0044282), tended to have lower mRNA levels in both Crohn's and ulcerative colitis (FDR < 1 × 10^6^; Supplementary Table 3). Although some of these pathway enrichment results might reflect the depletion in the relative frequency of colonic epithelial cells owing to immune cell infiltration in the inflamed sites, they are consistent with the decreased expression of PPARs as PPARs could regulate lipid signaling and metabolism^22^.

To replicate these observations, we used OMiCC to search for additional studies with ulcerative colitis but not Crohn's samples (Supplementary Note 3). The search returned four independent ulcerative colitis studies. Using these data, we formed four CGPs (Supplementary Table 1) and performed meta-analysis within OMiCC (Supplementary Table 4). The replication analysis showed a high concordance of the average fold-change (Supplementary Fig. 1; Pearson correlation = 0.85, *r*^*2*^ = 0.72, *P* < 2.2 × 10^−16^), a significant overlap in genes with increased or decreased expression (FDR < 0.05, Supplementary Fig. 2; 1,700 out of the 2,213 increased genes and 1,320 out of the 1,778 decreased genes were replicated (*P* = 0 for both replications, Fisher's exact test)); and a significant overlap in pathway enrichment results (Supplementary Fig. 3; for example, 1,451 of the 1,951 significant GO Biological Process terms enriched in the discovery meta-analysis for genes with increased expression had FDR < 0.05 in the validation set (*P* = 0, Fisher's exact test)). The specific findings from the discovery cohorts discussed above, including the observation that PPAR genes and genes in related pathways as well as those functioning in metabolic and catabolic processes had decreased expression in ulcerative colitis, were also replicated at the FDR < 0.05 level.

Thus, a meta-analysis of IBD using OMiCC revealed both known and potentially new differentially expressed genes and pathways. We have also demonstrated how performing additional meta-analyses using CGPs constructed from independent studies can be used to assess replicability. Similar analyses can be conducted using OMiCC for other biological phenotypes of interest.

## Supplementary Information

### Integrated supplementary information

Supplementary Figure 1 Scatter plot of the average fold-change of the discovery CGPs (x axis) vs. that of the validation CGPs (y axis) for UC. The correlation was assessed using a linear model (*r*^*2*^ = 0.72, *p* < 2.2×10^−16^); the fitted line is also shown.

Supplementary Figure 2 The degree of overlap in significantly increased (a) and decreased (b) genes between the discovery and validation meta-analyses. The significance of the overlaps was assessed using the GeneOverlap package (*p* = 0, Fisher's Exact Test).

Supplementary Figure 3 The degree of overlap in significant enriched gene sets between the discovery and validation meta-analyses. GO terms from the “Biological Processes” category enriched in genes with increased (a) or decreased (b) expression (*p* = 0 and 2.7×10^−204^, respectively; Fisher's Exact Test). Pathways (e.g., those from KEGG) enriched in genes with increased (c) and decreased (d) expression (*p* = 4.1×10^−184^ and *p* = 1.8×10^−143^, respectively; Fisher's Exact Test). We also tested enrichment and assessed overlap for the “Molecular Function” category from GO (*p* = 6×10^−107^ and *p* = 7.9×10^−51^ for genes with increased and decreased expression in UC, respectively) (Venn diagram not shown). The significance of the overlaps was assessed using the GeneOverlap package.

### Supplementary information

Supplementary Text and Figures Supplementary Figures 1–3 and Supplementary Notes 1–3 (PDF 1567 kb)

Supplementary Table 1 CGPs used in the meta-analyses of inflammatory bowel diseases use case (XLSX 10 kb)

Supplementary Table 2 Meta-analysis results of ulcerative colitis (UC) and Crohn's disease (CD) (output from OMiCC, which uses the RankProd1 package for computing these statistics). (XLSX 4485 kb)

Supplementary Table 3 Gene-set enrichment analysis results for genes whose expression was increasedor decreased in disease vs. healthy comparisons for ulcerative colitis and Crohn's disease (appear in different tabs of the Excel sheet). (XLSX 974 kb)

Supplementary Table 4 Meta-analysis results of validation UC CGPs constructed from independent studies using OMiCC. (XLSX 3527 kb)

## References

[1] Barrett T (2013). Nucleic Acids Res..

[2] Rustici G (2013). Nucleic Acids Res..

[3] Rung J, Brazma A (2013). Nat. Rev. Genet..

[4] Dudley JT, Deshpande T, Butte AJ (2011). Brief. Bioinform..

[5] Khatri P (2013). J. Exp. Med..

[6] Andres-Terre M (2015). Immunity.

[7] Sweeney TE, Shidham A, Wong HR, Khatri P (2015). Sci. Transl. Med..

[8] Rhodes DR (2004). Proc. Natl. Acad. Sci. USA.

[9] Ramasamy A, Mondry A, Holmes CC, Altman DG (2008). PLoS Med..

[10] Lowe HJ (1994). JAMA.

[11] Lamb J (2006). Science.

[12] Subramanian A (2005). Proc. Natl. Acad. Sci. USA.

[13] Reich M (2006). Nat. Genet..

[14] Gould, J. *GENE.E: Interact with GENE-E from R*. R package version 1.12.2 http://www.broadinstitute.org/cancer/software/GENE-E (2013).

[15] Huber W (2015). Nat. Methods.

[16] Bender R, Lange S (2001). J. Clin. Epidemiol..

[17] Leek JT (2010). Nat. Rev. Genet..

[18] Mayer L (2010). J. Gastroenterol..

[19] Van Beelen Granlund A (2013). PLoS One.

[20] Chen J, Bardes EE, Aronow BJ, Jegga AG (2009). Nucleic Acids Res..

[21] Dubuquoy L (2003). Gastroenterology.

[22] Wahli W, Michalik L (2012). Trends Endocrinol. Metab..

