# Supplementary figures and images for: A crowdsourcing approach for reusing and meta-analyzing gene expression data
Source: Nat Biotechnol. 2016 Jun 20;34(8):803–6. doi: 10.1038/nbt.3603 (PMC6871002; doi:10.1038/nbt.3603)

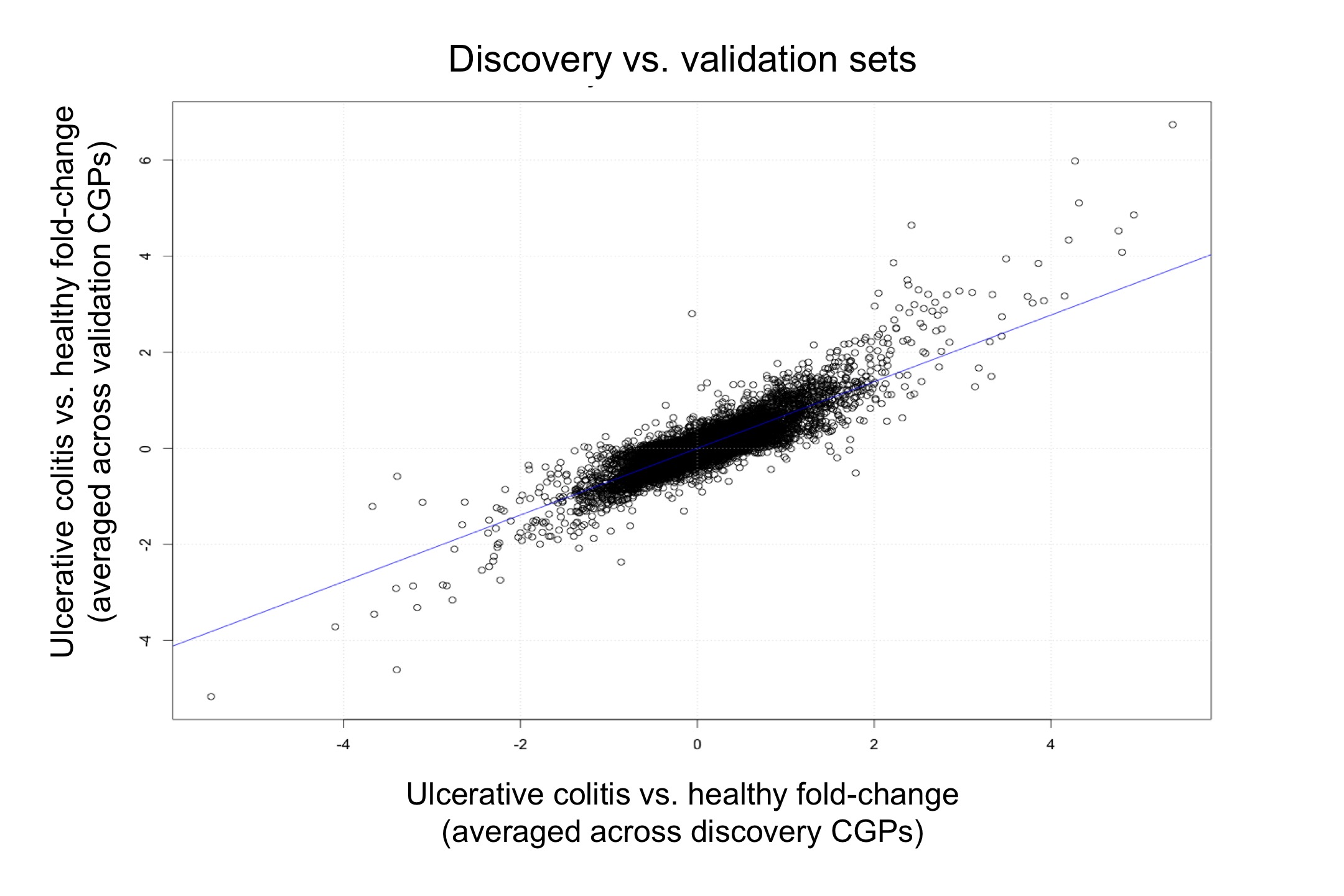

Supplement: Scatter plot of the average fold-change of the discovery CGPs (x axis) vs. that of the validation CGPs (y axis) for UC. — The correlation was assessed using a linear model (r2 = 0.72, p < 2.2×10^−16^); the fitted line is also shown. [file 41587_2016_Article_BFnbt3603_Fig2_ESM.jpg]

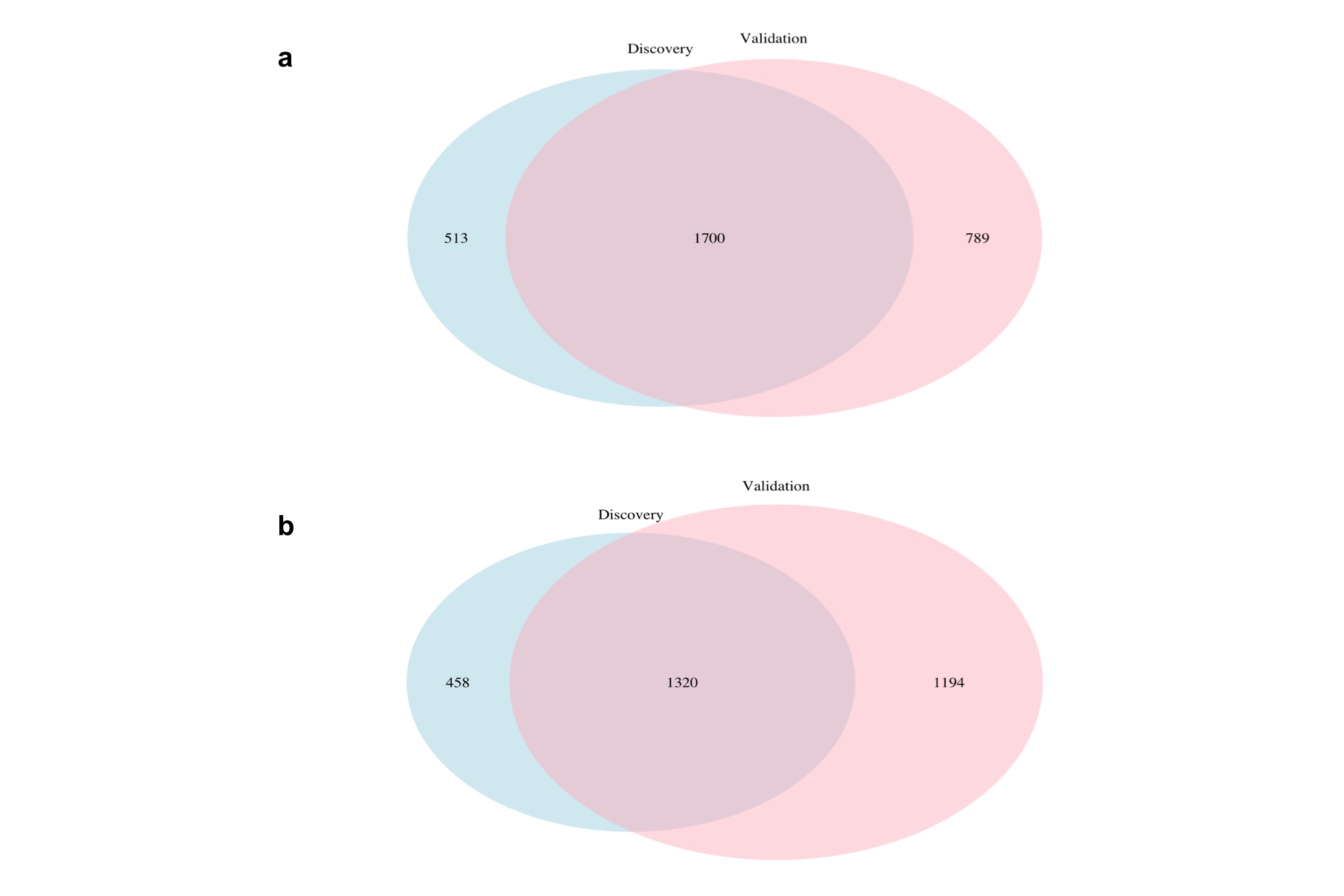

Supplement: The degree of overlap in significantly increased (a) and decreased (b) genes between the discovery and validation meta-analyses. — The significance of the overlaps was assessed using the GeneOverlap package (p = 0, Fisher's Exact Test). [file 41587_2016_Article_BFnbt3603_Fig3_ESM.jpg]

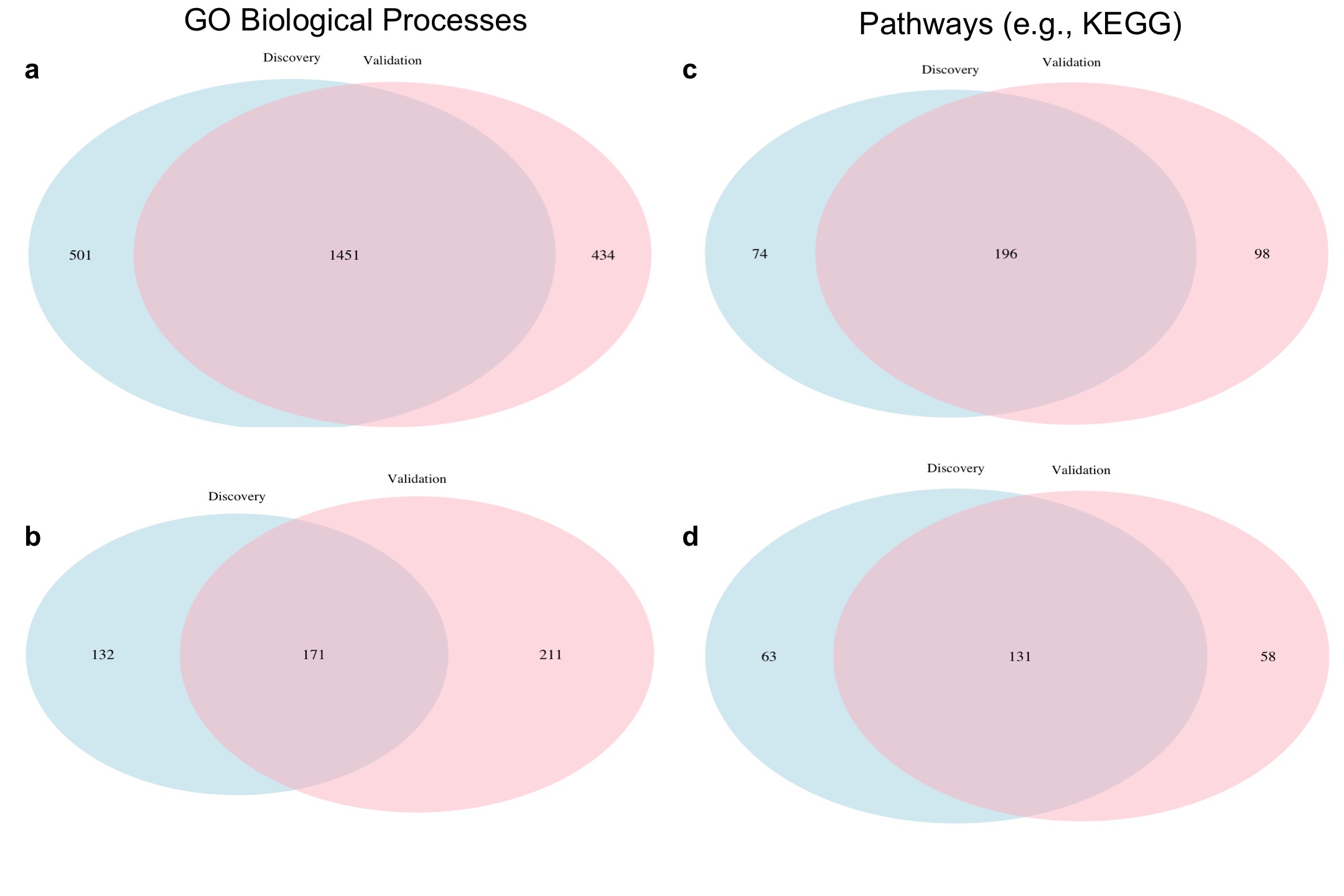

Supplement: The degree of overlap in significant enriched gene sets between the discovery and validation meta-analyses. — GO terms from the “Biological Processes” category enriched in genes with increased (a) or decreased (b) expression (p = 0 and 2.7×10−204, respectively; Fisher's Exact Test). Pathways (e.g., those from KEGG) enriched in genes with increased (c) and decreased (d) expression (p = 4.1×10−184 and p = 1.8×10−143, respectively; Fisher's Exact Test). We also tested enrichment and assessed overlap for the “Molecular Function” category from GO (p = 6×10−107 and p = 7.9×10^−51^ for genes with increased and decreased expression in UC, respectively) (Venn diagram not shown). The significance of the overlaps was assessed using the GeneOverlap package. [file 41587_2016_Article_BFnbt3603_Fig4_ESM.jpg]
