# Supplementary material for: A crowdsourcing approach for reusing and meta-analyzing gene expression data
Source: Nat Biotechnol. 2016 Jun 20;34(8):803–6. doi: 10.1038/nbt.3603 (PMC6871002; doi:10.1038/nbt.3603)
Supplement: Supplementary Text and Figures — Supplementary Figures 1–3 and Supplementary Notes 1–3 (PDF 1567 kb) [file 41587_2016_BFnbt3603_MOESM25_ESM.pdf]

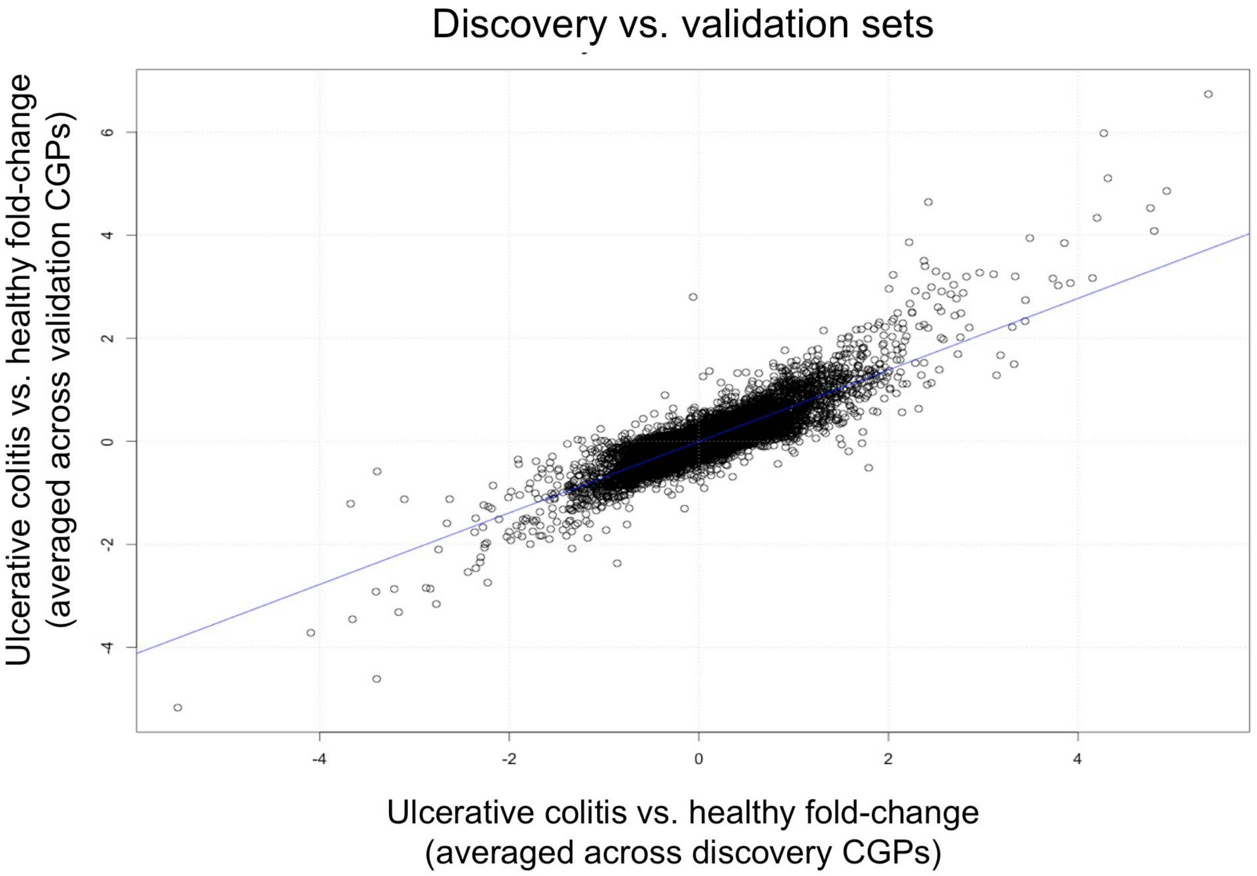

Supplementary Figure 1

Scatter plot of the average fold-change of the discovery CGPs (x axis) vs. that of the validation CGPs (y axis) for UC.

The correlation was assessed using a linear model ($r^2 = 0.72$, $p < 2.2\times10^{-16}$); the fitted line is also shown.

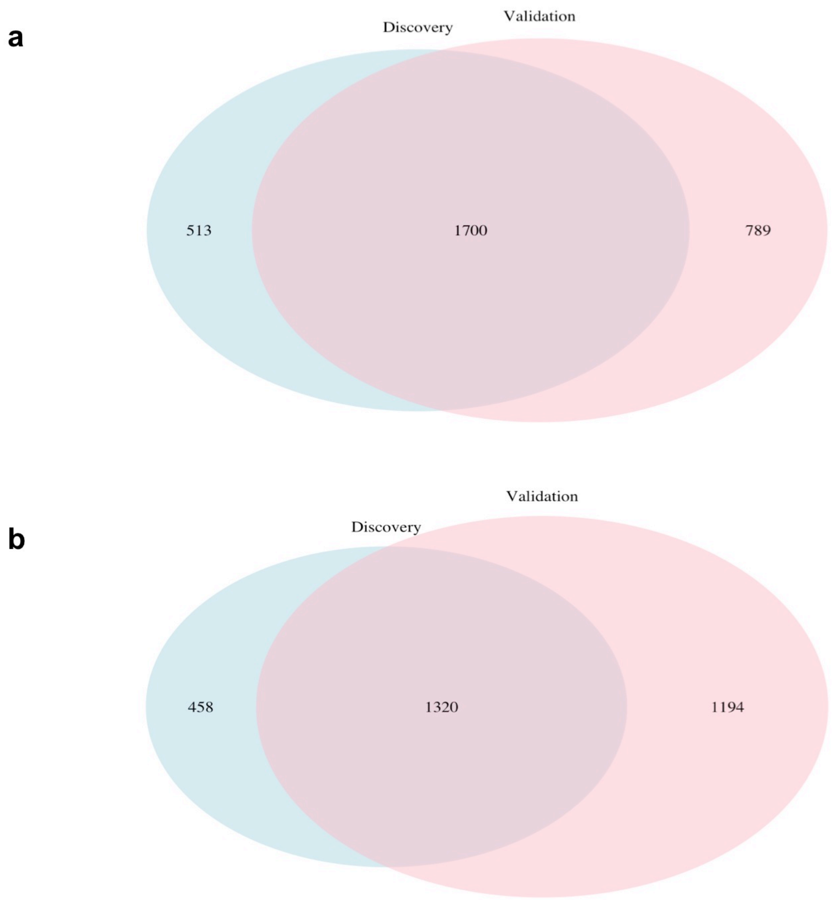

Supplementary Figure 2

The degree of overlap in significantly increased (a) and decreased (b) genes between the discovery and validation meta-analyses.

The significance of the overlaps was assessed using the GeneOverlap package (*p* = 0, Fisher's Exact Test).

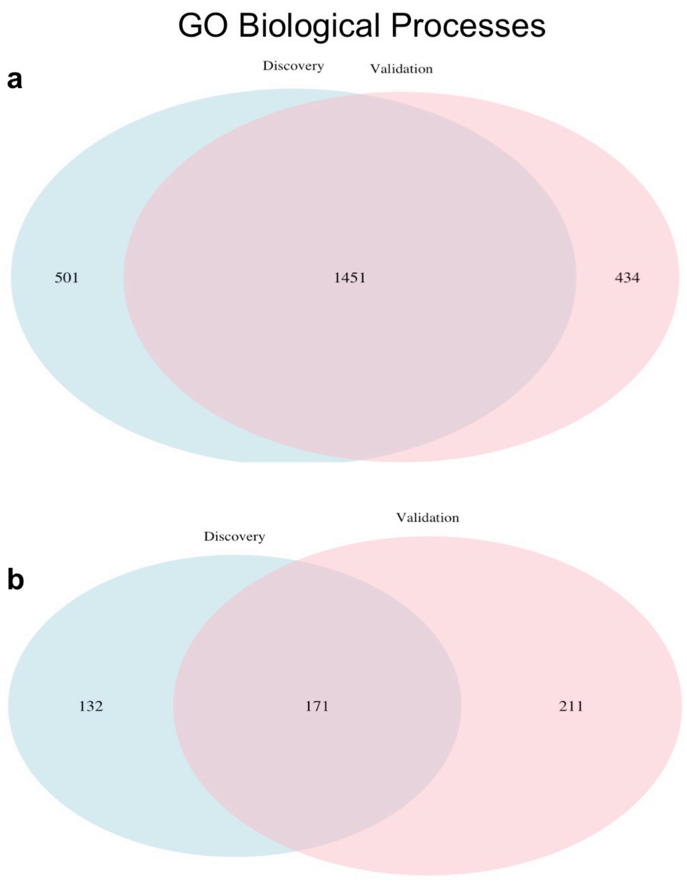

Supplementary Figure 3

The degree of overlap in significant enriched gene sets between the discovery and validation meta-analyses.

GO terms from the “Biological Processes” category enriched in genes with increased (a) or decreased (b) expression (p = 0 and $2.7\text{x}10^{-204}$, respectively; Fisher’s Exact Test). Pathways (e.g., those from KEGG) enriched in genes with increased (c) and decreased (d) expression (p = $4.1\text{x}10^{-184}$ and p = $1.8\text{x}10^{-143}$, respectively; Fisher’s Exact Test). We also tested enrichment and assessed overlap for the “Molecular Function” category from GO (p = $6\text{x}10^{-107}$ and p = $7.9\text{x}10^{-51}$ for genes with increased and decreased expression in UC, respectively) (Venn diagram not shown). The significance of the overlaps was assessed using the GeneOverlap package.

Supplementary Information

A crowdsourcing approach for reusing and meta-analyzing gene expression data across studies and platforms

Naisha Shah[1,*], Yongjian Guo[2,*], Katherine V. Wendelsdorf[1,*], Yong Lu[1], Rachel Sparks[1], John S. Tsang[1,#]

[1] Systems Genomics and Bioinformatics Unit and [2] Office of the Chief
Laboratory of Systems Biology, National Institute of Allergy and Infectious Diseases,
National Institutes of Health, Bethesda, Maryland, USA 20892

* These authors contributed equally to this work.

# Correspondence: JST (john.tsang@nih.gov)

List of Supplementary Tables (Excel tables)

Supplementary Table 1: CGPs used in the meta-analyses of inflammatory bowel diseases use case (this information was generated using OMiCC and pieced together in Excel – see **Supplementary Note 3**).

Supplementary Table 2: Meta-analysis results of ulcerative colitis (UC) and Crohn's disease (CD) (output from OMiCC, which uses the RankProd[1] package for computing these statistics). Note that the columns "pfp.DOWNregulated" and "pfp.UPregulated" are terminologies used by the RankProd package and they can be interpreted as the FDR for genes with increased or decreased expression in disease relative to healthy controls, respectively. Also see **Box 1** and **Supplementary Note 3**.

Supplementary Table 3: Gene-set enrichment analysis results for genes whose expression was increased or decreased in disease vs. healthy comparisons for ulcerative colitis and Crohn's disease (appear in different tabs of the Excel sheet). Analysis was performed separately for increased and decreased genes (DE genes determined using a FDR cutoff of 0.05 – see **Box 1** and **Supplementary Note 3**).

Supplementary Table 4: Meta-analysis results of validation UC CGPs constructed from independent studies using OMiCC. The first tab of the Excel sheet ("gene stats") contains the gene level statistics (output from OMiCC in same format as Supplementary Table 2). The "up genes pathway" and "down genes pathway" tabs contain the gene-set enrichment analysis results for up- and down-changing genes in UC vs. healthy comparisons. Analysis was performed separately for up- and down-changing genes (DE genes determined using a FDR cutoff of 0.05 – see **Box 1** and **Supplementary Note 3**).

Supplementary Note 1

Numerous useful resources have been created to enable the reuse and analysis of large-scale expression data, including 1) databases of manually-curated expression data (e.g., GEO DataSet[2] and NextBio[3]), 2) tools for data retrieval (e.g., MaRe[4]), sample annotation, and data collation (e.g., InSilicoDB[5]), 3) software for data analysis and visualization (e.g., GenePattern[6]), and 4) packages for meta-analysis of multiple data sets (e.g., INMEX[7]). Despite these advances, these tools tend to focus on one or a subset of steps and thus forming complete workflows requires additional programming. While there has been active commercial developments, including a platform called NextBio[8] that offers more turnkey solutions, its contents and annotations were pre-compiled using proprietary algorithms, and thus customization of analysis parameters, formation of new signatures, and using data sets not already available within their platform can be difficult, if not impossible. Another commercial platform called InsilicoDB[5] offers interfaces for annotating, collating, and analyzing both user-supplied and public data. However, it only allows a limited number of free analyses using public data sets (10 as of December 2015). In general, a fee-based service could limit the size and diversity of the user community; for example, less well-funded groups and research areas, as well as organizations from developing countries tend to have less access.

Supplementary Note 2: Software and Data Processing Procedures

OMiCC software framework

OMiCC includes backend data-processing pipelines, a MySQL database (database schema available upon request), and a web server serving the web user interface (UI). The backend data-processing pipelines were mainly developed using the BPipe package[9]. These pipelines were used for retrieving data from GEO[2], performing data normalization, formatting data output and updating the contents of the MySQL database. The pipelines were run on a Linux based computer cluster through the Sun Grid Engine.

The OMiCC database is updated and accessed by the backend data processing pipelines and web server through Hibernate, an object-relational mapping library that facilitates efficient data processing.

The OMiCC web server was developed using the Grails framework and is run on a Tomcat 7 web container. The Grails searchable plugin was used to index and search GEO data records through a Lucene indexing engine to further improve performance. The front-end web interface was developed using the JQuery library. The OMiCC data analysis jobs are executed using R on an Rserve server.

Gene-expression data and pre-processing

More than 26,000 human and mouse gene expression studies and the associated meta-data were downloaded from GEO capturing ~90% of the data deposited on or before June 2015 (not all data are covered because certain technology platforms are excluded; see below). In the future, our plan is to update OMiCC with new GEO releases once every 6 months. However, we plan to retrieve data at least 6 months old to avoid using newly deposited data since those tend to be updated frequently after initial deposition. Up to three types of expression data are made available to OMiCC users depending on the platform and data availability in GEO: 1) for Affymetrix platforms, RMA normalized data derived from the raw CEL files using the Affymetrix power tools package[10]; 2) GEO

series matrix data sets (i.e., GEO user-submitted versions of the data); and 3) quantile normalized versions of (2) - normalization was performed using the preprocessCore R package[11]. GEO encourages submission of Minimum Information About a Microarray Experiment (MIAME) compliant data, which requires submission of both raw and normalized data files. However, a substantial proportion of the GEO studies still do not have associated raw data files available. In addition, not all studies or the associated publication, if there is one, contain sufficient information on the process used to generate the normalized data. Thus, in addition to providing the GEO user-submitted normalized data, we also provide a quantile-normalized version of that data processed using our own procedure. In OMiCC, the choice of which normalized data to use, if more than one are available, is dependent on issues related to, for example, potential batch effects and technical artifacts of individual studies. Under the assumption that the user-submitted normalized files in GEO (i.e., the GEO series matrix data) were processed appropriately and because such files are available for almost all studies, by default OMiCC uses the quantile-normalized version of the GEO series matrix data. However, the user can choose other normalized file(s) for performing analyses within OMiCC.

Quality control assessment was performed on all data sets using arrayQualityMetrics R package[12]. Samples flagged as outliers by all three outlier-detection methods in the package were removed. The metrics used by these outlier detection methods were: 1) distances between arrays using mean absolute difference, 2) signal intensity distributions of the arrays by computing the Kolmogorov-Smirnov statistic, and 3) individual array quality using Hoeffding's D-statistic[12].

We also provide probe-to-HUGO gene symbol mapping for more than 1900 GEO experimental platforms (GPLs[2]) covering ~90% of the samples in our database. For a given platform, we first attempted to obtain the mapping from the manufacturer's website; if it was not available, we retrieved it either from the AILUN database[13] or from the corresponding GPL record in GEO[2].

Nature Biotechnology: doi:10.1038/nbt.3603

Construction of comparison group pairs (CGPs)

Gene expression studies and data sets from GEO can be searched and browsed using the OMiCC web interface. For querying, the US National Library of Medicine's controlled vocabulary MeSH (Medical Subject Headings) database[14] is used to facilitate searching using synonymous terms. OMiCC can be used to create groups of samples from a study. A Comparison Group Pair (CGP) consists of two sample groups, e.g., perturbed sample group and control/unperturbed sample group (Fig. 1b). Once CGPs are created, they can be collated and differential expression profiles (DEPs – see below) can be created automatically using OMiCC. Both sample groups and CGPs can be shared with other OMiCC users by flagging them as "public"; these can then be searched and used by other OMiCC users. Each sample group can be annotated using 6 concepts, namely *perturbation* (e.g., IL-4 treatment), *time with perturbation*, *disease*, *sample type* (e.g., monocytes), *sample source* (e.g., blood) and *other* (to capture free text and additional annotation categories). We use MeSH terms to assist with the annotation process to better categorize the samples and to facilitate structured searches by other users. We also allow free-text for annotation, especially in cases where MeSH does not contain the appropriate tag terms. Similar to the study search interface, OMiCC also facilitates searches on publically available groups of samples and CGPs.

Gene-expression analysis

OMiCC can perform two main types of analyses given a CGP: 1) Differential expression analysis to derive a differential expression profile (DEP) – i.e., the magnitude and statistical significance of gene-expression differences between the two groups within the CGP for all genes, and significantly differentially expressed (DE) genes based on a statistical cutoff threshold; and 2) Meta-analysis of more than one CGP within the same or across different studies and experimental platforms (Fig. 1a). OMiCC can compute DEP statistics using several different methods: 1) Linear modeling using the *Limma* R package[15], 2) Mann-Whitney test[16] , and 3) Student's t-test[17]. When samples across the two groups within a CGP are paired (i.e. individual samples with condition 1 have a corresponding

Nature Biotechnology: doi:10.1038/nbt.3603

matching samples in condition 2, e.g., the paired samples come from the same subject over two time-points), a paired statistical test is performed. By default, OMiCC selects the widely used Limma (Linear Models for Microarray data)[18] method to calculate expression differences between two sample groups in a CGP. Limma has been shown to perform better especially when working with small sample sizes[19]. The Limma R package provides several methods for adjusting the p-values to account for multiple hypothesis testing. We support the same set of multiple testing correction methods for Limma, as well as for the other two options in OMiCC (Mann-Whitney and Student's t-tests) using the *p.adjust* function in R. A list of significant DE genes is generated using a cutoff threshold on the resulting p-values or the adjusted p-values (at the user's discretion).

DEPs from selected CGPs within a compendium are merged into a gene/probe-by-CGP matrix file and can be downloaded for down-steam analysis. The user has the option of selecting different types of statistics for creating the data matrix such as t-statistic and B-values from *Limma*. In addition, one can visualize hierarchical clustering of DEPs and the 500 most varying genes within the data matrix by downloading a heatmap created using the *pheatmap* R package[20].

Meta-analysis in OMiCC is performed using the *RankProd* R package[1]. It is a non-parametric method and can achieve higher sensitivity and specificity compared to other meta-analysis methods according to Hong *et al.*[21]. RankProd first converts fold-change values derived from normalized expression data within a CGP into ranks. Then, a rank product is calculated for each gene across the CGPs. Lastly, meta-p value and false discovery rate (called "percentage of false positive predictions" in RankProd) are calculated based on permuted expression values. See the original RandProd publication referenced above for further details.

To support cross-platform analyses, DEP creation, collation, and meta-analyses can be performed at the gene (HUGO symbols) level whenever probe-to-gene mapping information is available (See "Gene-expression data and pre-processing" in Methods). Promiscuous probes, i.e., those that mapped to multiple gene symbols, were removed from further analysis. For gene

Nature Biotechnology: doi:10.1038/nbt.3603

symbols covered by multiple probes, we adopted the commonly used approach of taking the median across these probes[22].

OMiCC output, visualization and supported down-stream analysis tools

When a DEP analysis is performed on CGP(s), OMiCC provides, for each CGP: 1) statistics describing differential expression for all genes or probes (depending on the user's selection of working at the probe or gene level); 2) a list of differentially expressed (DE) genes based on a default or a user-provided p-value cutoff threshold; 3) heatmap showing normalized expression values for the 100 most significant DE genes; 4) files with expression (GCT format) and phenotype labels (CLS format); and 5) a Java Network Launch Protocol (JNLP) file that launches GENE-E[23] pre-loaded with data.

A file with the list of DE genes (item 2 above) can be uploaded to tools such as Database for Annotation, Visualization and Integrated Discovery (DAVID)[24]. GCT and CLS formatted files (4) can be used in tools such as Gene Set Enrichment Analysis[25] and GenePattern[6]. Such files are provided for the convenience of the user to perform additional down-stream analyses using existing tools.

In addition, if DEP analysis is performed on multiple CGPs, a DEP matrix file is provided containing common genes or probes across all CGPs as rows and CGPs as columns. The exported values (e.g. –log10(p value) or T-statistic) in the file can be selected by the user via the OMiCC interface. With the DEP matrix file, OMiCC also outputs two types of plots: 1) heatmap with clustering of CGPs containg the 500 most variable genes or probes across all CGPs, and 2) barplot showing number of DE genes per CGP. These visualizations can be used for diagnostics, e.g., identification of outlier CGPs (e.g.: a CGP with no DE genes could indicate technical artifact or low-sample size). A CGP information file can also be downloaded.

When a meta-analysis is performed on CGPs, two files are generated for download: 1) a file containing statistics such as the meta- p-value, -false discovery rate and -fold change for each gene or probe (output from RankProd R package[1]); 2) a list of DE genes based on a default or a user-

Nature Biotechnology: doi:10.1038/nbt.3603

provided p-value cutoff threshold; and 3) a file containing information for all CGPs, such as the number of samples and annotation tags.

Please refer to the tutorial page on the OMiCC website for additional details (https://omicc.niaid.nih.gov/help/index).

Supplementary Note 3: Meta-analysis of IBD

We conducted the analysis when OMiCC contained data deposited to GEO on or before January 2014 (OMiCC currently covers up to June 2015; see **Supplementary Note 2** on OMiCC's data update approach). We used OMiCC's search interface to query for human IBD studies using the term "Crohn's" with the aim of finding studies containing both disease subtypes (CD and UC). Based on the information provided in OMiCC and in the original paper of each study, we limited studies to those containing tissue samples from CD, UC and healthy subjects in the same study. For simplicity, we restricted microarray platforms to two versions of the most widely used platforms from Affymetrix (Affymetrix Human Genome U133 Plus 2.0 Array, and Affymetrix GeneChip Human Genome U133 Plus 2.0 Array)—OMiCC allows user to filter on microarray platforms in a search; OMiCC also indicates which platforms are the most widely used ones to help guide user to select the platforms that are likely the most robust. Note that OMiCC contains pre-processed data from and supports a large number of platforms, not only Affymetrix.

We next created CGPs from studies found using these criteria (**Supplementary Table 1**). We performed two meta-analyses at the gene level using "Normalized GEO Data" (see **Supplementary Note 2** on the different types of data): 1) for all CD versus healthy CGPs, and 2) for all UC versus healthy CGPs. Significantly differentially expressed genes with a "percentage of false positive predictions" (PFP: a false discovery rate (FDR) equivalent metric from the RankProd[1] meta-analysis package used in OMiCC) ("Adjusted P value" in OMiCC) of less than 0.05 were identified within OMiCC. The meta-analysis results were downloaded from OMiCC for down-stream gene-set enrichment analysis using the ToppGene Suite software[26], separately for the up- and down-changing genes in disease vs. healthy subjects. The meta-analysis results can be accessed at the following OMiCC analysis results page:

UC: https://omicc.niaid.nih.gov/myProject/showResult/824?ac=E6Y727OKG

CD: https://omicc.niaid.nih.gov/myProject/showResult/839?ac=RFSLX7RK4

For our UC validation meta-analysis, we queried human datasets in OMiCC using the term "Ulcerative Colitis". Studies were limited to those that contained samples from subjects with UC, but not CD samples (assessing CD replication using meta-analysis was not possible as only one additional CD study was found using the same search criteria we used for UC). From this list of studies, we selected the same platforms we used in our "discovery" analysis (Affymetrix Human Genome U133 Plus 2.0 Array, Affymetrix GeneChip Human Genome U133 Plus 2.0 Array). We then eliminated any studies in which the RNA was not derived from tissue biopsy. Next, studies that did not contain both affected subjects and control subjects were removed. Finally, we evaluated the primary literature of each study and eliminated any studies in which we were unsure if samples may have been duplicated in another candidate study. This included studies in which the publications had shared authors or originated from the same research institution. In some instances, the authors of the respective publications were contacted to clarify if some samples may have been duplicated. The meta-analysis results can be accessed at the following OMiCC analysis results page (also see **Supplementary Table 4**):

https://omicc.niaid.nih.gov/myProject/showResult/837?ac=O7NQ5LMDM

We imported data from Supplementary Tables 2-4 into R to assess replication between discovery and validation CGP sets. **Supplementary Figures 1-3** were generated in R (The "VennDiagram" package[27] was used to generate Venn Diagrams). We used the GeneOverlap package to compute Fisher's Exact Test p values on overlaps at the gene and gene set/pathway levels (https://www.bioconductor.org/packages/release/bioc/html/GeneOverlap.html). Correlation in Supplementary Fig. 1 was assessed using a linear model. R code will be available upon request.

Nature Biotechnology: doi:10.1038/nbt.3603

References

1. Hong, F. *et al.* RankProd: a bioconductor package for detecting differentially expressed genes in meta-analysis. *Bioinformatics* **22,** 2825–7 (2006).
2. Barrett, T. *et al.* NCBI GEO: archive for functional genomics data sets–update. *Nucleic Acids Res.* **41,** D991–5 (2013).
3. Kupershmidt, I. *et al.* Ontology-Based Meta-Analysis of Global Collections of High-Throughput Public Data. *PLoS One* **5,** 13 (2010).
4. Ivliev, A. E., 't Hoen, P. A. C., Villerius, M. P., den Dunnen, J. T. & Brandt, B. W. Microarray retriever: a web-based tool for searching and large scale retrieval of public microarray data. *Nucleic Acids Res.* **36,** W327–31 (2008).
5. Coletta, A. *et al.* InSilico DB genomic datasets hub: an efficient starting point for analyzing genome-wide studies in GenePattern, Integrative Genomics Viewer, and R/Bioconductor. *Genome Biol.* **13,** R104 (2012).
6. Reich, M. *et al.* GenePattern 2.0. *Nat. Genet.* **38,** 500–1 (2006).
7. Xia, J. *et al.* INMEX–a web-based tool for integrative meta-analysis of expression data. *Nucleic Acids Res.* **41,** W63–70 (2013).
8. Kupershmidt, I. *et al.* Ontology-Based Meta-Analysis of Global Collections of High-Throughput Public Data. *PLoS One* **5,** 13 (2010).
9. Sadedin, S. P., Pope, B. & Oshlack, A. Bpipe: a tool for running and managing bioinformatics pipelines. *Bioinformatics* **28,** 1525–6 (2012).
10. Affymetrix Power Tools. Affymetrix Power Tools.
11. Bolstad, B. M., Irizarry, R. ., Astrand, M. & Speed, T. P. A comparison of normalization methods for high density oligonucleotide array data based on variance and bias. *Bioinformatics* **19,** 185–193 (2003).
12. Kauffmann, A., Gentleman, R. & Huber, W. arrayQualityMetrics–a bioconductor package for

Nature Biotechnology: doi:10.1038/nbt.3603

quality assessment of microarray data. *Bioinformatics* **25,** 415–416 (2009).

13. Chen, R., Li, L. & Butte, A. J. AILUN: reannotating gene expression data automatically. *Nat. Methods* **4,** 879 (2007).

14. ROGERS, F. B. Medical subject headings. *Bull. Med. Libr. Assoc.* **51,** 114–6 (1963).

15. Smyth, G. K. in (eds. Gentleman, R., Carey, V., Dudoit, S., Irizarry, R. & Huber, W.) 397—–420 (Springer, 2005).

16. Mann, H. B. & Whitney, D. R. On a Test of Whether one of Two Random Variables is Stochastically Larger than the Other. *Ann. Math. Stat.* **18,** 50–60 (1947).

17. Student. THE PROBABLE ERROR OF A MEAN. *Biometrika* **6,** 1–25 (1908).

18. Smyth, G. K. Linear models and empirical bayes methods for assessing differential expression in microarray experiments. *Stat. Appl. Genet. Mol. Biol.* **3,** Article3 (2004).

19. Jeanmougin, M. *et al.* Should we abandon the t-test in the analysis of gene expression microarray data: a comparison of variance modeling strategies. *PLoS One* **5,** e12336 (2010).

20. Kolde, R. pheatmap: Pretty Heatmaps. (2013).

21. Hong, F. & Breitling, R. A comparison of meta-analysis methods for detecting differentially expressed genes in microarray experiments. *Bioinformatics* **24,** 374–82 (2008).

22. Hughey, J. J. & Butte, A. J. Robust meta-analysis of gene expression using the elastic net. *Nucleic Acids Res.* gkv229 (2015). doi:10.1093/nar/gkv229

23. Gould, J. Interactive exploration of matrices in GENE-E.

24. Huang, D. W., Sherman, B. T. & Lempicki, R. A. Systematic and integrative analysis of large gene lists using DAVID bioinformatics resources. *Nat. Protoc.* **4,** 44–57 (2009).

25. Subramanian, A. *et al.* Gene set enrichment analysis: a knowledge-based approach for interpreting genome-wide expression profiles. *Proc. Natl. Acad. Sci. U. S. A.* **102,** 15545–50 (2005).

26. Chen, J., Bardes, E. E., Aronow, B. J. & Jegga, A. G. ToppGene Suite for gene list enrichment

analysis and candidate gene prioritization. *Nucleic Acids Res.* **37,** W305–11 (2009).

27. Chen, H. & Boutros, P. C. VennDiagram: a package for the generation of highly-customizable Venn and Euler diagrams in R. *BMC Bioinformatics* **12,** 35 (2011).
